# Vertex Displacement-Based Discontinuous Deformation Analysis Using Virtual Element Method

**DOI:** 10.3390/ma14051252

**Published:** 2021-03-06

**Authors:** Hongming Luo, Guanhua Sun, Lipeng Liu, Wei Jiang

**Affiliations:** 1 State Key Laboratory of Geomechanics and Geotechnical Engineering, Institute of Rock and Soil Mechanics, Chinese Academy of Sciences, Wuhan 430071, China; hmluo@whrsm.ac.cn (H.L.); ghsun@whrsm.ac.cn (G.S.); 2 State Key Laboratory of Simulation and Regulation of Water Cycle in River Basin, China Institute of Water Resources and Hydropower Research, Beijing 100038, China; liulip@iwhr.com; 3 Key Laboratory of Geological Hazards on Three Gorges Reservoir Area, Ministry of Education, China Three Gorges University, Yichang 443002, China

**Keywords:** DDA, VEM, degrees of freedom, projection

## Abstract

To avoid disadvantages caused by rotational degrees of freedom in the original Discontinuous Deformation Analysis (DDA), a new block displacement mode is defined within a time step, where displacements of all the block vertices are taken as the degrees of freedom. An individual virtual element space ***V***_1_(Ω) is defined for a block to illustrate displacement of the block using the Virtual Element Method (VEM). Based on VEM theory, the total potential energy of the block system in DDA is formulated and minimized to obtain the global equilibrium equations. At the end of a time step, the vertex coordinates are updated by adding their incremental displacement to their previous coordinates. In the new method, no explicit expression for the displacement ***u*** is required, and all numerical integrations can be easily computed. Four numerical examples originally designed by Shi are analyzed, verifying the effectiveness and precision of the proposed method.

## 1. Introduction

Discontinuous Deformation Analysis (DDA) [1], a novel numerical method for analyzing the dynamic mechanical behavior of a block system in cases of large displacement, was verified as an effective tool in solving a variety of discontinuities in rock problems [2,3]. In rock-mass engineering, DDA has been employed to handle a great deal of problems, e.g., landslide process simulations [4,5], slope stability assessments [6,7], blasting effect evaluation [8], crack propagation simulation [9,10], seismic wave propagation analysis [11], and rock burst prediction [12]. Applications of 2D DDA in the modeling of rock-mass dynamics until 2017 were summarized by Ning [13].

Numerous enhancements have been put forward to improve the performance of traditional DDA. To alleviate the sensibility of penalty parameters, contacts between the blocks were remodeled using the Lagrange multiplier method [14], Augmented Lagrangian method [15], Complementary theory [16], and Variational Inequality theory [17]. Apart from modifying kinetic velocity using the dynamic factor [18], the damping effect was used to reflect the energy dissipation by imposing viscous boundary conditions [19] and by adding viscous forces into the equilibrium equation [20]. A couple of strategies, such as adopting a higher-order displacement function [21] and partitioning blocks using finite element mesh [22], were introduced to acquire a more detailed stress field in each block. Moreover, many efforts were made to develop a 3D version of DDA [23,24,25,26], considering that 3D DDA is preferred for problems in practical engineering. The biggest bottleneck to establishing a robust 3D version is the lack of an excellent contact theory [27]. Shi [28] proposed the entrance block theory to facilitate contact treatment in 3D DDA. In addition, some efforts, such as GPU-based parallel computation [29] and explicit computation [30], were made to improve the computational efficiency when simulating large-scale problems.

Within a time step, the degrees of freedom ***d*** in the traditional DDA were defined for a block by six independent variables, which consists of two incremental rigid translations, one incremental rigid rotation angle, and three incremental constant strain components. For a point ***x*** in the block, the incremental displacement ***u*** is calculated as the product of ***d*** and the transformation matrix ***T***; then, the global equilibrium equation is derived from this displacement function. The displacement function uses the first-order approximation of sin *r_0_* = *r_0_* and cos *r_0_* = 1 for a small rotation angle *r_0_*, which induces accumulated errors over the time steps and sometimes causes issues. The most noticeable issue is the unreasonable volume expansion when a large rotation occurs. To restrain this issue, some scholars proposed various emendations [31,32,33], in which the post-adjustment strategy was most popular in the existing DDA codes [34,35]. This method, albeit simple and effective, resulted in a displacement different from the displacement evaluated in the equilibrium equation. Even with a tiny difference, the contact state might be entirely changed for a contact pair. Then, to simulate the continuous–discontinuous deformation, numerical methods for the continuum and non-continuum models were increasingly coupled. DDA was coupled with finite element method (FEM) by some scholars [9,22] for simulation of a rock failure process. In classical FEM, displacements at the element nodes are chosen as the degrees of freedom. This inconsistency in the degrees of freedom between DDA and FEM causes a barrier in developing a compact and efficient code for the coupling method.

In a recent study [36], the displacement of blocks in DDA was reformulated by using Wachspress interpolation to achieve a higher-order stress distribution within blocks. This approach selected displacements at the vertices as the degrees of freedom for a block. In this study, a DDA with such new degrees of freedom is called a vertex displacement-based DDA. This method was improved using the Polygonal Finite Element method (PFEM). Besides Wachspress interpolation [37], a series of interpolations [38,39,40] were proposed to construct the displacement functions for a polygonal element with more than four nodes in PFEM. By taking displacements at the vertices as the degrees of freedom, these displacement models can avoid demerits caused by the degrees of freedom in an original DDA. However, because the rational function is involved in the shape function, the theoretical derivations and calculations of the stiffness and mass matrix are quite complicated and it is hard to ensure precision of the numerical integration [41,42].

In 2013, the Virtual Element Method (VEM) was proposed to handle the very general polygons, dispensing sophisticated integrations on the element [43,44,45,46]. VEM was considered the evolution of the mimetic finite difference method and a generalization of FEM. For flexibility with regard to mesh generation and element shapes, VEM has become a hot topic in numerical methods since it was proposed [47,48,49,50,51,52]. In this study, a new vertex displacement-based DDA is developed using the strength of VEM. Defining the degrees of freedom by using the incremental displacements ***u*** at the vertices of a block, an individual virtual element space ***V***_1_(Ω) is adopted to describe the displacements of points in the block, and the projector Π*_P_**u*** from ***V***_1_(Ω) on the linear displacement space ***P***_1_(Ω) is deduced. Next, the total potential energy is investigated for the block system. In the potential energy, the bilinear forms of ***u*** are expressed as the summation of the exact solution of Π*_P_**u*** and an approximation of ***u***-Π*_P_**u***. The potential energy induced by the contact restraints are derived using the new degrees of freedom. Then, for the block system, the global equilibrium equation is derived based on the principle of minimum potential energy. Finally, the open–close iteration strategy is employed to resolve the global equilibrium equation as the original DDA. The proposed method avoids the issues attributable to first-order approximation for a small rotation angle *r*_0_ in the original DDA and has a higher computational efficiency than vertex displacement-based DDA using displacement functions in PFEM. The validity and effectiveness of the proposed method are verified by several numerical examples.

## 2. Basic Principles of DDA

**Figure 1:**
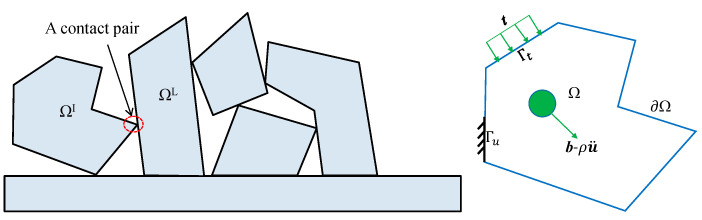
A typical block system and an individual block in Discontinuous Deformation Analysis (DDA).

As Figure 1 shows, a DDA block system always consists of n_b_ discrete blocks with their individual domains and boundaries. The domain Ω^I^ of block I is bounded by ∂Ω^I^, which is usually composed of the Dirichlet boundary $\Gamma_{u}^{I}$ and the traction boundary $\Gamma_{t}^{I}$. The displacement on $\Gamma_{u}^{I}$ is prescribed as ***û***^I^, and the surface traction on $\Gamma_{t}^{I}$ is denoted by ***t***^I^. Here, the superscript I is the block index. The deformations and large displacements of a block in DDA are accumulated by the incremental displacements and deformations over time steps. The displacements of blocks are independent from each other, and contact constraints are imposed on the interactions between blocks.

Within a time step, DDA requires the incremental displacements u = (***u***^1^, ***u***^2^, …, ***u***^nb^) to be the unique minimizer of the total potential energy of the block system:

(1) $$u=\underset{v\in V}{\arg\min}J\left(v\right),\;J\left(v\right)=\frac{1}{2}\alpha \left(v,v\right)-f\left(v\right)+J^{\prime }\left(v\right)$$

in which ***V*** is the incremental displacement space of the block system:

(2) $$\;V=V^{1}\times V^{2}\times \ldots \times V^{n_{b}},\;\mathrm{with}\;V^{I}=\left\{v^{I}\in {\left[H^{1}\left(\Omega^{I}\right)\right]}^{2}:v^{I}{|}_{\Gamma_{u}^{I}}={\hat{u}}^{I}\right\}$$

where ***J***’(v) represents the energy due to the contact constraints. A key feature of DDA is that rigorous contact constraints are used to manage the interactions between blocks. For the contact pair marked in Figure 1, ***J***’(v) induced by the contact constraints is determined by ***u***^I^∈***V^I^*** and ***u***^L^∈***V^L^.***

The bilinear form α(u, v) and the linear form *f*(v) are computed for u∈***V*** and v∈***V*** by

(3) $$\alpha \left(u,v\right)=\sum_{I=1}^{n_{b}}\alpha \left(u^{I},v^{I}\right),f\left(v\right)=\sum_{I=1}^{n_{b}}f\left(v^{I}\right)$$

because all blocks are physically isolated in DDA. For ease of description, the superscript indicating blocks is omitted unless otherwise noted. The bilinear form *α* (***u***, ***v***) for ***u***∈***V*** and ***v***∈***V*** represents the energy due to elastic deformation and the inertial force:

(4) $$\alpha \left(u,v\right)=\int_{\Omega }\sigma \left(u\right):\varepsilon \left(v\right)\;dx+\int_{\Omega }\rho \ddot{u}\cdot v\;dx$$

where ***ε*** is the incremental constant strain decided by ***u***:

(5) $$\varepsilon \left(u\right)=\frac{1}{2}\left(\nabla u+\nabla^{T}u\right)$$

in which ∇ is the gradient operator. ***σ*** is the incremental Cauchy stress related to ***ε*** by the constitutive equation:

(6) σ(u)=D[ε(u)]

where *D* is a constant elasticity tensor. *ρ* is the mass per unit area. For dynamic analysis, the inertial force is essential in DDA. New-mark time integration scheme is adopted in the original DDA, and the acceleration is assumed to be constant within a time step. Denoting by ***V*_0_** the velocity of block I at the origin of a time step and by ∆, the time interval of the time step, the acceleration is computed as

(7) $$\ddot{u}=\frac{2}{\Delta^{2}}u-\frac{2}{\Delta }V_{0}$$

The linear form *f*(***v***) is defined by

(8) $$f\left(v\right)=\int_{\Omega }b\cdot vdx-\int_{\Omega }\sigma_{0}:\varepsilon \left(v\right)dx+\int_{\Omega }t\cdot vds$$

where ***b*** denotes the constant body force and ***σ***_0_ is the constant stress accumulated over the previous time steps.

When the displacement function is determined for a block with the associated degrees of freedom, Equation (1) induces the global equilibrium equations with the following matrix formulation for the block system.

(9) $$\left[\begin{array}{ccccc} K_{11} & K_{12} & K_{13} & \ldots & K_{1\left(n_{b}\right)}\\ K_{21} & K_{22} & K_{23} & \ldots & K_{2\left(n_{b}\right)}\\ K_{31} & K_{32} & K_{33} & \ldots & K_{3\left(n_{b}\right)}\\ \vdots & \vdots & \vdots & \ddots & \vdots \\ K_{\left(n_{b}\right)1} & K_{\left(n_{b}\right)2} & K_{\left(n_{b}\right)3} & \ldots & K_{\left(n_{b}\right)\left(n_{b}\right)} \end{array}\right]\left[\begin{array}{c} d^{1}\\ d^{2}\\ d^{3}\\ \vdots \\ d^{n_{b}} \end{array}\right]=\left[\begin{array}{c} F^{1}\\ F^{2}\\ F^{3}\\ \vdots \\ F^{n_{b}} \end{array}\right]$$

in which ***d****^I^* is the degrees of freedom concerning block *I*. ***F****^I^* is the generalized load vector with the same dimension of ***d****^I^*. Denoting the dimension of ***d****^I^* with dim(***d****^I^*), ***K****_II_* is a dim(***d****^I^*) × dim(***d****^I^*) matrix and ***K****_I__L(I≠__L)_* is a dim(***d****^I^*) × dim(***d****^L^*) matrix denoting the contact restraints on blocks *I* and *L*. If no contact pair is provided by blocks *I* and *L* in the current time step, ***K****_I__L_* is zero.

## 3. Demerits Caused by the Original Degrees of Freedom and Previous Attempts to Construct a Vertex Displacement-Based DDA

### 3.1. Demerits Caused by the Original Degrees of Freedom in DDA

For block Ω, the traditional DDA defines the degrees of freedom ***d*** within one time step:

(10) $$d={\left(\begin{array}{cccccc} u_{0} & v_{0} & r_{0} & \varepsilon_{x} & \varepsilon_{y} & \gamma_{xy} \end{array}\right)}^{T}$$

where *u*_0_ and *v*_0_ represent the increments of the rigid horizontal and vertical translations, respectively; *r*_0_ denotes the increment of the rigid rotation angle around the central point (*x*_0_, *y*_0_) of block Ω; and (*ε_x_*, *ε*_y_, *γ_xy_*) denotes the increments of the constant strains. Under small deformation assumption, the increments of the displacement ***u*** *= (u, v)^T^* at a point ***x*** = (*x*, *y*) in block Ω are computed as

(11) u=Td 

and ***T*** is the translation matrix as follows:

(12) $$T=\left[\begin{array}{cccccc} 1 & 0 & -\left(y-y_{o}\right) & \left(x-x_{o}\right) & 0 & 0.5\left(y-y_{o}\right)\\ 0 & 1 & \left(x-x_{o}\right) & 0 & \left(y-y_{o}\right) & 0.5\left(x-x_{o}\right) \end{array}\right]$$

Equation (11) is a variation of a standard first-order displacement function [1].

The displacement caused by rigid movement and the deformation of the block constitute the displacement of a block under small deformation assumption. Therefore, the displacement function for finite rotation should be

(13) $$\begin{array}{l} u=u_{0}+\left(x-x_{0}\right)\left(\cos r_{0}-1\right)-\left(y-y_{0}\right)\sin r_{0}+\left(x-x_{0}\right)\varepsilon_{x}+0.5\left(y-y_{0}\right)\gamma_{xy}\\ v=v_{0}+\left(x-x_{0}\right)\sin r_{0}+\left(y-y_{0}\right)\left(\cos r_{0}-1\right)+\left(y-y_{0}\right)\varepsilon_{y}+0.5\left(x-x_{0}\right)\gamma_{xy} \end{array}$$

Comparing Equation (13) with Equation (11), the approximations of cos *r*_0_ = 1 and sin *r*_0_ = *r*_0_ are adopted for the small rotation angle *r_0_* in Equation (11). The approximation errors accumulated over time steps can lead to false volume expansion when a large rotation occurs. Various modifications were suggested to remedy this defect, in which the post-adjustment strategy is most popular in the existing DDA codes. After vector ***d*** is obtained, the post-adjustment strategy employs Equation (13) to calculate the incremental displacement ***u***. Although simple and effective in most cases, the resulting displacement must be different from the displacement estimated in the equilibrium equation. Sometimes, for a contact pair, the state adopted in the equilibrium equation may be not coincident with the geometry relationship of the resulted configurations. An example is provided in Section 5.1 to demonstrate this issue.

Besides that, it is noticed that DDA was coupled with a range of numerical methods for continuum models to simulate the continuous–discontinuous deformation, e.g., crack propagation [9,22]. Most popular numerical methods in terms of continuum mechanics select the displacements at the nodes of an element as the degrees of freedom. DDA defines the degrees of freedom using the constant strain, the rigid translation, and the rigid rotation referring to the centroid of a block. The difference in the degrees of freedom causes a barrier in developing a compact code when coupling DDA to other numerical tools.

### 3.2. Previous Attempts to Construct Vertex Displacement-Based DDA

By taking the increments of the displacements at the vertices as the degrees of freedom, vertex displacement-based DDA can remedy the demerits induced by the original degrees of freedom. Supposing that a block Ω has *n* vertices, the incremental displacements ***u****_i_*= (*u_i_*, *v_i_*)*^T^* at the vertex ***x****_i_* constitute the new degrees of freedom:

(14) $$\bar{d}={\left(\begin{array}{ccccccc} u_{1} & v_{1} & u_{2} & v_{2} & \ldots & u_{n} & v_{n} \end{array}\right)}^{T}$$

In this way, the penetration value of a contact pair in the equilibrium equations is directly calculated within a time step and the coordinates of the vertices are updated by directly adding the incremental displacements to their previous coordinates, inducing penetrations in the equilibrium equation and allowing the updated configurations to coincide.

By using the new degrees of freedom, the increment ***u*** of the displacement at a point ***x*** in a block Ω can be computed in a similar method to FEM, that is

(15) $$u=\left(\begin{array}{c} u\\ v \end{array}\right)=\left(\begin{array}{cccccccc} N_{1} & 0 & N_{2} & 0 & \ldots & \ldots & N_{n} & 0\\ 0 & N_{1} & 0 & N_{2} & \ldots & \ldots & 0 & N_{n} \end{array}\right)\bar{d}=N\bar{d}$$

where *N_i_* is the shape function in terms of the vertex ***x****_i_*. Because the block in DDA might have more than four vertices, the shape functions developed in PFEM were introduced to construct *N_i_* [36]. In the recent study [36], Wachspress interpolation was selected as the shape function for developing vertex displacement-based DDA.

**Figure 2:**
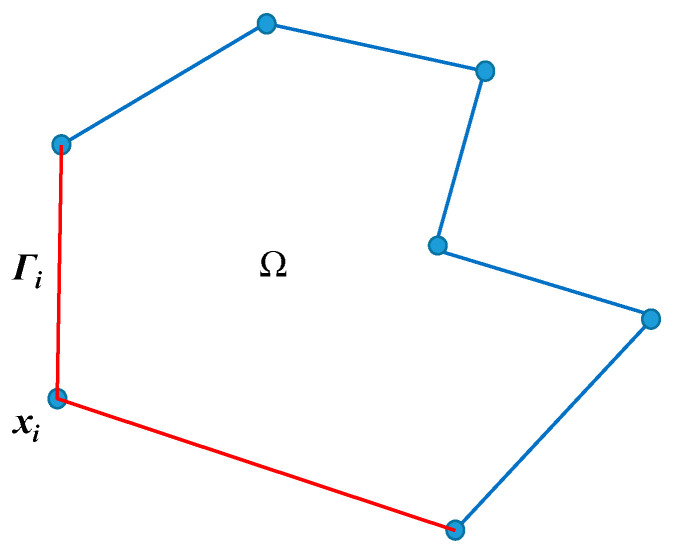
A polygon and its boundary division.

Sukumar and MalsCh concluded the properties required for *N_i_* to develop an effective PFEM [38]. As shown in Figure 2, a block Ω has *n* vertices located at ***x****_i_* and its boundary is composed of *n* straight edges. The red edges with vertex ***x****_i_* are denoted by *Γ_i_*. *N_i_* should be satisfied.

(1) Partition of unity, boundedness, and nonnegativity:

(16) $$\sum {}_{i=1}^{n}N_{i}\left(x\right)=1,0\leq N_{i}\left(x\right)\leq 1$$

(2) Kronecker-delta property:

(17) $$\;N_{i}\left(x_{j}\right)=\delta_{ij}$$

(3) Linear completeness:

(18) $$\sum {}_{i=1}^{n}N_{i}\left(x\right)\;x_{i}=x$$

(4) With *C*^∞^ being within block Ω while *C*^0^ is on the boundary, *N_i_* must be piece-wise linear along *Γ_i_* but vanishes at the other edges. This property ensures that the boundary of a straight line is still a straight line after deformation, which benefits contact detection and contact condition imposition.

In the last two decades, generalized barycentric coordinates were successfully adopted as the shape function in PFEM. A series of generalized barycentric coordinates, such as Wachspress interpolation [37], maximum entropy interpolation [38], mean value coordinates [39] and Harmonic coordinates [40], were developed, marking remarkable progress in the theory and application of PFEM.

**Figure 3:**
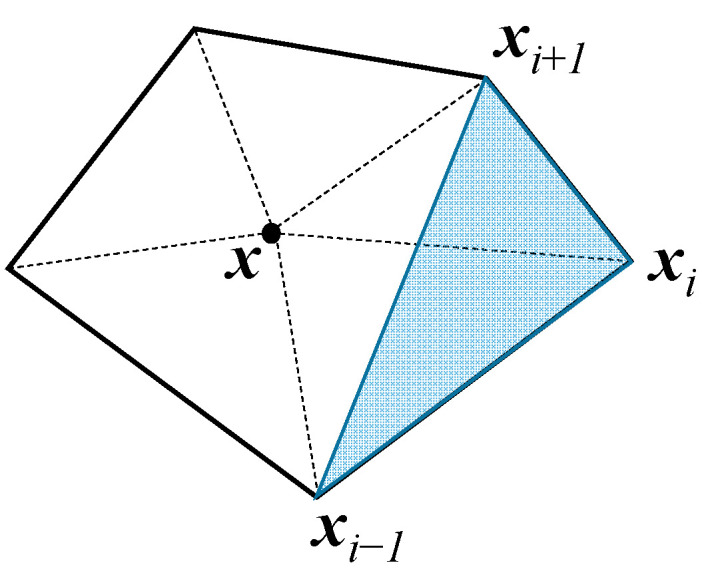
Triangles involved in the calculation of N_i_ using Wachspress interpolation.

Take Wachspress interpolation for example; the shape function *N_i_* is defined for a point ***x*** within a polygon as follows.

(19) $$N_{i}\left(x\right)=\frac{A\left(x_{i-1},x_{i},x_{i+1}\right)\left(\underset{k\neq i,i-1}{\Pi }A\left(x,x_{k},x_{k+1}\right)\right)}{\sum_{j=1}^{n}A\left(x_{j-1},x_{j},x_{j+1}\right)\left(\underset{k\neq j,j-1}{\Pi }A\left(x,x_{k},x_{k+1}\right)\right)}$$

where ***x***_1_, ***x***_2_, …, ***x***_n_ are the vertices of the polygon arranged counterclockwise and *A*(***x***_i_, ***x***_j_, ***x***_k_) represents the area of a triangle connecting three points, such as in Figure 3. By observation, the basic principles required for *N_i_* are completely satisfied by Definition (19).

However, the definitions of *N_i_* in PFEM are the rational functions, which make the derivation and integration process more cumbersome when computing the block matrices. Though some enhancements [38] were made to simplify the formulas of *N_i_*, the rational function is indispensable to definite *N_i_*, which creates difficulties in the integration over a polygon. Up until now, the most popular way to solve this issue was partitioning the polygon into subdomains with certain shapes. Then, the Hammer or Gauss integration scheme was employed to compute the integration on each sub-triangle or sub-rectangle. The summation of the integrations on subdomains was considered the integration over the original polygon. While the integration error was considerable, some remedies were proposed to address the integration error for PFEM [42]. In conclusion, the additional work in the derivation and programming is very expensive when using the interpolation function *N_i_* in PFEM. When Equation (15) is adopted to govern the block displacement, some advantages of the original DDA can compromised.

## 4. Vertex Displacement-Based DDA Using VEM

### 4.1. Virtual Element Spaces

VEM [43,44,45,46] provides a new way to construct a vertex displacement-based DDA. Because DDA blocks are physically isolated, an individual virtual element space ***V****_k_*(Ω) can be defined for a block as follows:

(20) $$\;V_{k}\left(\Omega \right)=\left\{v\in {\left[H^{1}\left(\Omega \right)\cap C^{0}\left(\partial \Omega \right)\right]}^{2}:\Delta v\in {\left[P_{k-2}\left(\Omega \right)\right]}^{2},v_{|\Gamma }\in {\left[P_{k}\left(\Gamma \right)\right]}^{2}\forall \Gamma \in \partial \Omega \right\},$$

in which *Γ* denotes a edge of Ω, *k* ≥ 1 is a fixed integer index indicating the order of accuracy of the approach, and *P_k_*(Ω) is the space of polynomials of degree less than or equal to *k* in Ω. Obviously, [*P_k_*(Ω)]^2^⊆***V***_k_(Ω). For simplicity, [*P_k_*(Ω)]^2^ is denoted as ***P***_k_(Ω).

We take *k* = 1 in this study. There are three main reasons for this choice: (i) The degrees of freedom for ***V***_1_(Ω) only involve the values of ***v*** at the vertices of Ω, in accordance with the new degrees of freedom defined in Equation (14). (ii) The incremental displacements are expected to be piece-wise linear at the block boundary, which is satisfied by ***V***_1_(Ω). (iii) In the view that the original DDA adopted a complete first-order displacement function, the degree of accuracy is not threatened by using ***V***_1_(Ω). Therefore, an individual virtual element space ***V***_1_(Ω) is defined for a block, and the associated degrees of freedom are the new degrees of freedom defined in Equation (14). The dimension of the space ***V***_1_(Ω) is 2n, where *n* denotes the number of vertices for the block.

Provided that there are n_b_ blocks in the block system, the total virtual element space can be given as

(21) $$\;V_{1}=V_{1}^{1}\left(\Omega^{1}\right)\times V_{1}^{2}\left(\Omega^{2}\right)\times \ldots \times V_{1}^{n_{b}}\left(\Omega^{n_{b}}\right),$$

and the total degrees of freedom comprise the increments of displacements at the vertices of all blocks. As a result, the solution space ***V*** in Equation (1) is replaced by ***V***_1_.

### 4.2. The Projection Operator Π_P_**u**: **V**_1_(Ω)→**P**_1_(Ω)

To minimize the total potential energy of the block system, the computation of *α* (***u***, ***v***) from ***V***_1_(Ω) × ***V***_1_(Ω) to *R* defined in Equation (4) is inevitable, which can be accomplished in the framework of VEM theory. Referring to [47], two projectors Π^∇^***u****:* ***V***_1_(Ω)→***P***_1_(Ω) and Π^0^***u****:* ***V***_1_(Ω)→***P***_1_(Ω) are respectively defined using the orthogonality conditions:

(22) $$\int_{\Omega }\nabla \left(\Pi^{\nabla }u\right)\cdot \nabla p\;\mathrm{dx}=\int_{\Omega }\nabla u\cdot \nabla p\;\mathrm{dx}\;\forall p\in P_{1}\left(\Omega \right),$$

and

(23) $$\int_{\Omega }\left(\Pi^{0}u\right)\cdot p\;\mathrm{dx}=\int_{\Omega }u\cdot p\;\mathrm{dx}\;\forall p\in P_{1}\left(\Omega \right).$$

where Π^0^***u*** cannot be obtained from (23) because the zero- and first-order moments of ***u*** in Ω are unknown. To solve this issue, an additional property stating that the moments of order 0 and 1 of ***u*** and Π^∇^***u*** coincide was added by B. Ahmad [44] to the space ***V***_1_(Ω), resulting in the projection operator of Π^0^***u*** being the same as the projection operator of Π^∇^***u***. For simplicity, Π_P_***u*** is adopted to express Π^∇^***u*** and Π^0^***u*** uniformly. In this study, Π_P_***u*** is deduced using an approach different from the standard procedure [46].

Beforehand, two notations are defined. For a function ***w***, the average of the values it assumes at the vertices of Ω is denoted by ***w***^∗^:

(24) $$w^{*}=\frac{1}{n}\sum_{i=1}^{n}w\left(x_{i}\right),$$

and the volume average of ***w*** over Ω is denoted by ⟨***w***⟩:

(25) $$\langle w\rangle =\frac{1}{S}\int_{\Omega }w\;dx.$$

where *S* is the area of Ω.

***P***_1_(Ω) is the space of linear displacements over Ω, which can be split into two displacement spaces due to rigid motions and constant strain. We denote the space regarding rigid motions by ***R***(Ω) and the space concerning constant strain by ***C***(Ω):

(26) $$R\left(\Omega \right)=\left\{a+B_{R}\left(x-x^{*}\right):a\in R^{2},B_{R}\in R^{2\times 2},B_{R}^{T}=-B_{R}\right\},$$

(27) $$C\left(\Omega \right)=\left\{B_{C}\left(x-x^{*}\right):B_{C}\in R^{2\times 2},B_{C}^{T}=B_{C}\right\}.$$

Obviously, ***P***_1_(Ω) is the summation of ***R***(Ω) and ***C***(Ω). Both ***R***(Ω) and ***C***(Ω) are subspaces of ***V***_1_(Ω). Two projection maps, Π_R_: ***V***_1_(Ω)→***R***(Ω) and Π_C_: ***V***_1_(Ω)→***C***(Ω), are defined to extract the rigid motions and constant strain of ***u*∊*V***_1_(Ω). Considering that elements of ***C***(Ω) should contain no rigid motion and that elements of ***R***(Ω) should contain no constant strain, the following orthogonality conditions are imposed on the two maps:

(28) $$\Pi_{R}c=0,\;\forall c\in C\left(\Omega \right),$$

(29) $$\Pi_{C}r=0,\;\forall r\in R\left(\Omega \right).$$

Gain et al. [48] proved that projection maps Π_R_ and Π_C_ satisfying the above properties can be given by

(30) $$\Pi_{R}u=u^{*}+\langle \omega \left(u\right)\rangle \left(x-x^{*}\right)\;\mathrm{with}\;\omega \left(u\right)=\frac{1}{2}\left(\nabla u-\nabla^{T}u\right)$$

and

(31) $$\Pi_{C}u=\langle \varepsilon \left(u\right)\rangle \left(x-x^{*}\right)\;$$

Due to the orthogonality conditions in Equations (28) and (29), we have

(32) $$\Pi_{P}u=\Pi_{R}u+\Pi_{C}u=u^{*}+\langle \nabla u\rangle \left(x-x^{*}\right).$$

It is noticed that

(33) $$\int_{\Omega }\nabla \left(\Pi_{P}u\right)\;dx=\int_{\Omega }\langle \nabla u\rangle \;dx=\;\int_{\Omega }\nabla u\;dx,$$

and that ∇***p*** in Equation (22) contains only constant elements**.** Obviously, the projection map Π_P_***u*** given by Equation (32) satisfies Equation (22).

**Figure 4:**
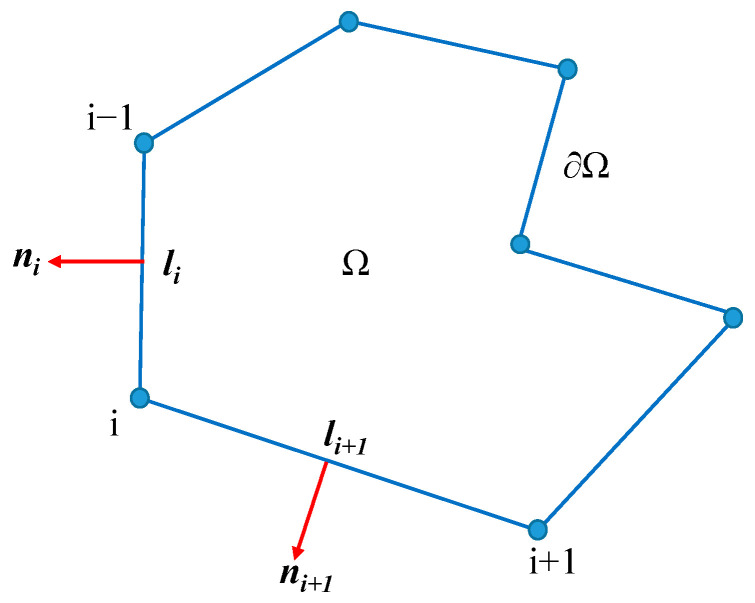
The notation li and ***n**_i_* in ***H***.

Using the integration by parts, the areal integrals in Equation (32) can easily be converted to the boundary integrals, and then, it can be computed precisely because ***u*** = (*u*, *v*) is linear on each edge of Ω. Denoting the length of edge *i* connecting vertex *i*−1 and vertex *i* as *l_i_*, as Figure 4 shows, and the outward unit normal vector on edge *i* as ***n****_i_* = (*n_ix_*, *n_iy_*)*^T^*, 〈∇***u***〉 can be computed as

(34) $$\begin{array}{l} \langle \nabla u\rangle =\frac{1}{S}\int_{\Omega }\nabla u\;dx=\frac{1}{S}\left[\begin{array}{cc} \int_{\partial \Omega }un_{x}\;ds & \int_{\partial \Omega }un_{y}\;ds\\ \int_{\partial \Omega }vn_{x}\;ds & \int_{\partial \Omega }vn_{y}\;ds \end{array}\right]\\ =\frac{1}{S}\left[\begin{array}{cc} \sum_{i=1}^{n}\frac{l_{i}n_{ix}+l_{i+1}n_{\left(i+1\right)x}}{2}u_{i} & \sum_{i=1}^{n}\frac{l_{i}n_{iy}+l_{i+1}n_{\left(i+1\right)y}}{2}u_{i}\\ \sum_{i=1}^{n}\frac{l_{i}n_{ix}+l_{i+1}n_{\left(i+1\right)x}}{2}v_{i} & \sum_{i=1}^{n}\frac{l_{i}n_{iy}+l_{i+1}n_{\left(i+1\right)y}}{2}v_{i} \end{array}\right] \end{array}$$

By substituting Equation (34) into Equation (32), we have

(35) $$\Pi_{P}u=\frac{1}{n}\left(\begin{array}{c} \sum_{i=1}^{n}u_{i}\\ \sum_{i=1}^{n}v_{i} \end{array}\right)+\frac{1}{S}\left[\begin{array}{cc} \sum_{i=1}^{n}\frac{l_{i}n_{ix}+l_{i+1}n_{\left(i+1\right)x}}{2}u_{i} & \sum_{i=1}^{n}\frac{l_{i}n_{iy}+l_{i+1}n_{\left(i+1\right)y}}{2}u_{i}\\ \sum_{i=1}^{n}\frac{l_{i}n_{ix}+l_{i+1}n_{\left(i+1\right)x}}{2}v_{i} & \sum_{i=1}^{n}\frac{l_{i}n_{iy}+l_{i+1}n_{\left(i+1\right)y}}{2}v_{i} \end{array}\right]\left(\begin{array}{c} x-x^{*}\\ y-y^{*} \end{array}\right)$$

For ease of subsequent performance, another matrix expression of Π_P_***u*** is given as

(36) $$\Pi_{P}u=T^{*}H\bar{d},$$

in which

(37) $$T^{*}=\left[\begin{array}{cccccc} 1 & 0 & -\left(y-y^{*}\right) & \left(x-x^{*}\right) & 0 & 0.5\left(y-y^{*}\right)\\ 0 & 1 & \left(x-x^{*}\right) & 0 & \left(y-y^{*}\right) & 0.5\left(x-x^{*}\right) \end{array}\right]$$

and

(38) $$\;H=\left(\begin{array}{ccccccc} \frac{1}{n} & 0 & \frac{1}{n} & 0 & \ldots & \frac{1}{n} & 0\\ 0 & \frac{1}{n} & 0 & \frac{1}{n} & \ldots & 0 & \frac{1}{n}\\ \frac{l_{1}n_{1y}+l_{2}n_{2y}}{-4S} & \frac{l_{1}n_{1x}+l_{2}n_{2x}}{4S} & \frac{l_{2}n_{2y}+l_{3}n_{3y}}{-4S} & \frac{l_{2}n_{2x}+l_{3}n_{3x}}{4S} & \ldots & \frac{l_{n}n_{ny}+l_{1}n_{1y}}{-4S} & \frac{l_{n}n_{nx}+l_{1}n_{1x}}{4S}\\ \frac{l_{1}n_{1x}+l_{2}n_{2x}}{2S} & 0 & \frac{l_{2}n_{2x}+l_{3}n_{3x}}{2S} & 0 & \ldots & \frac{l_{n}n_{nx}+l_{1}n_{1x}}{2S} & 0\\ 0 & \frac{l_{1}n_{1y}+l_{2}n_{2y}}{2S} & 0 & \frac{l_{2}n_{2y}+l_{3}n_{3y}}{2S} & \ldots & 0 & \frac{l_{n}n_{ny}+l_{1}n_{1y}}{2S}\\ \frac{l_{1}n_{1y}+l_{2}n_{2y}}{2S} & \frac{l_{1}n_{1x}+l_{2}n_{2x}}{2S} & \frac{l_{2}n_{2y}+l_{3}n_{3y}}{2S} & \frac{l_{2}n_{2x}+l_{3}n_{3x}}{2S} & \ldots & \frac{l_{n}n_{ny}+l_{1}n_{1y}}{2S} & \frac{l_{n}n_{nx}+l_{1}n_{1x}}{2S} \end{array}\right).$$

Due to the orthogonality conditions in Equations (28) and (29), the constant strain tensor ***ε***(Π_P_***u***) can be computed as follows:

(39) $$\begin{array}{l} \varepsilon \left(\Pi_{P}u\right)=\varepsilon \left(\Pi_{C}u\right)=\langle \varepsilon \left(u\right)\rangle \\ =\frac{1}{S}\left[\begin{array}{cc} \sum_{i=1}^{n}\frac{l_{i}n_{ix}+l_{i+1}n_{\left(i+1\right)x}}{2}u_{i} & \sum_{i=1}^{n}\left(\frac{l_{i}n_{iy}+l_{i+1}n_{\left(i+1\right)y}}{4}u_{i}++\frac{l_{i}n_{ix}+l_{i+1}n_{\left(i+1\right)x}}{4}v_{i}\right)\\ \sum_{i=1}^{n}\left(\frac{l_{i}n_{iy}+l_{i+1}n_{\left(i+1\right)y}}{4}u_{i}++\frac{l_{i}n_{ix}+l_{i+1}n_{\left(i+1\right)x}}{4}v_{i}\right) & \sum_{i=1}^{n}\frac{l_{i}n_{iy}+l_{i+1}n_{\left(i+1\right)y}}{2}v_{i} \end{array}\right] \end{array}$$

and a matrix expression of the constant strain vector ***ε*** = (*ε_x_*, *ε*_y_, *γ_xy_*) can be given as

(40) $$\varepsilon \left(\Pi_{P}u\right)=\frac{1}{S}\left[\begin{array}{c} \sum_{i=1}^{n}\frac{l_{i}n_{ix}+l_{i+1}n_{\left(i+1\right)x}}{2}u_{i}\\ \sum_{i=1}^{n}\frac{l_{i}n_{iy}+l_{i+1}n_{\left(i+1\right)y}}{2}v_{i}\\ \sum_{i=1}^{n}\left(\frac{l_{i}n_{iy}+l_{i+1}n_{\left(i+1\right)y}}{2}u_{i}++\frac{l_{i}n_{ix}+l_{i+1}n_{\left(i+1\right)x}}{2}v_{i}\right) \end{array}\right]=P_{C}\bar{d}$$

in which

(41) $$P_{c}=\left(\begin{array}{ccccccc} \frac{l_{1}n_{1x}+l_{2}n_{2x}}{2S} & 0 & \frac{l_{2}n_{2x}+l_{3}n_{3x}}{2S} & 0 & \ldots & \frac{l_{n}n_{nx}+l_{1}n_{1x}}{2S} & 0\\ 0 & \frac{l_{1}n_{1y}+l_{2}n_{2y}}{2S} & 0 & \frac{l_{2}n_{2y}+l_{3}n_{3y}}{2S} & \ldots & 0 & \frac{l_{n}n_{ny}+l_{1}n_{1y}}{2S}\\ \frac{l_{1}n_{1y}+l_{2}n_{2y}}{2S} & \frac{l_{1}n_{1x}+l_{2}n_{2x}}{2S} & \frac{l_{2}n_{2y}+l_{3}n_{3y}}{2S} & \frac{l_{2}n_{2x}+l_{3}n_{3x}}{2S} & \ldots & \frac{l_{n}n_{ny}+l_{1}n_{1y}}{2S} & \frac{l_{n}n_{nx}+l_{1}n_{1x}}{2S} \end{array}\right).$$

Obviously, ***P***_c_ is actually composed by the latter three rows in ***H***.

An element ***u***∈***V***_1_(Ω) can be decomposed into Π_P_***u*** and the residual item ***u***−Π_P_***u***. Then, a bilinear form from ***V***_1_(Ω) × ***V***_1_(Ω) to *R* can be analyzed without the explicit expression of ***u***, which will be demonstrated in the next section.

### 4.3. Computation of α(**u**, **v**) and f(**v**)

For simplicity of expression, *α* (***u***, ***v***) in Equation (4) and *f*(***v***) in Equation (8) are rewritten as

(42) $$\alpha \left(u,v\right)=\alpha^{E}+\alpha^{M}-\alpha^{V},f\left(v\right)=f^{b}+f^{t}-f^{\sigma }$$

in which

(43) $$\alpha^{E}=\int_{\Omega }\varepsilon^{T}\left(v\right)\sigma \left(u\right)\;dx,\;\alpha^{M}=\frac{2\rho }{\Delta^{2}}\int_{\Omega }v^{T}u\;dx,\;\alpha^{V}=\frac{2\rho }{\Delta }\int_{\Omega }v^{T}V_{0}\;dx$$

and

(44) $$f^{b}=\int_{\Omega }v^{T}b\;dx,\;f^{t}=\int_{\partial \Omega }v^{T}tds,\;f^{\sigma }=\int_{\Omega }\varepsilon^{T}\left(v\right)\sigma_{0}dx$$

It is noticed that, in this study, ***V***_0_ is considered an element in ***V***_1_(Ω) to estimate ***V***_0_ within the block because ***V***_0_ at the block vertices are directly related to ***u***. Consequently, *α^V^* is a bilinear form. ***V***_0_ can also be evaluated using the rigid body motion and the constant strain associated with Π_P_***u***. That will induce ***V***_0_ to become an element in ***P_1_***(Ω) and *α^V^* to become a linear form.

The bilinear forms are formulated first. Due to the orthogonality conditions (22) and (23), any bilinear form *α*(***u***, ***v***) regarding two elements ***u***, ***v***∈***V***_1_(Ω) can be rewritten as

(45) $$\alpha \left(u,v\right)=a\left(\Pi_{P}u,\Pi_{P}v\right)+a\left(u-\Pi_{P}u,v-\Pi_{P}v\right).$$

On the right side of Equation (45), the first term named the consistency term can be precisely calculated while the second term called the stabilization term represents the contribution of the residual item ***u***−Π_P_***u*** to the bilinear form. The stabilization term cannot produce an exact solution, so the second term in VEM is usually denoted as *s*(***u***−Π_P_***u***, ***v***−Π_P_***v***) to indicate that it is an approximation. The standard scheme of *s*(***u***−Π_P_***u***, ***v***−Π_P_***v***) was provided in classical VEM literature [46], but it is quite free in the construction of *s*(***u***−Π_P_***u***, ***v***−Π_P_***v***) in practice [48,49]. The stabilization term proposed by Veiga [46] is adopted in this study:

(46) $$s\left(u-\Pi_{P}u,v-\Pi_{P}v\right)=\sum_{i=1}^{n}\eta \left[u\left(x_{i}\right)-\Pi_{P}u\left(x_{i}\right)\right]\left[v\left(x_{i}\right)-\Pi_{P}v\left(x_{i}\right)\right]$$

where *η* is a positive parameter ensuring the right scaling of the bilinear form assigned to the residual item.

Using Equation (45), the bilinear form *α^E^* is rewritten as follows:

(47) $$\alpha^{E}\left(u,u\right)=\alpha^{E}\left(\Pi_{P}u,\Pi_{P}u\right)+s^{E}\left(u-\Pi_{P}u,u-\Pi_{P}u\right)$$

Substituting the matrix expression (40) regarding ***ε***(Π_P_***u***) into Equation (47), the consistency term is

(48) $$\alpha^{E}\left(\Pi_{P}u,\Pi_{P}u\right)=\int_{\Omega }\varepsilon^{T}\left(\Pi_{P}u\right)\sigma \left(\Pi u\right)\;dx={\bar{d}}^{T}\left[SP_{c}^{T}DP_{c}\right]\bar{d}$$

in which ***D*** is the elastic matrix

The stabilization term in Equation (47) is

(49) $$s^{E}\left(u-\Pi_{P}u,u-\Pi_{P}u\right)=\sum_{i=1}^{n}\eta^{E}{\left[u\left(x_{i}\right)-\Pi_{P}u\left(x_{i}\right)\right]}^{2}={\bar{d}}^{T}\left[\eta^{E}{\left(I-P_{u}\right)}^{T}\left(I-P_{u}\right)\right]\bar{d}$$

where ***I*** is a 2*n* × 2*n* unit matrix and ***P****_u_* is a 2*n* × 2*n* constant matrix:

(50) $$P_{u}=\left[\begin{array}{c} T^{*}\left(x_{1}\right)\\ T^{*}\left(x_{2}\right)\\ \vdots \\ T^{*}\left(x_{n}\right) \end{array}\right]H$$

In the view that *s*^E^ is the approximation of *α^E^*, the positive parameters *η^E^* can be decided upon by requiring that *s*^E^ and *α^E^* are comparable. The consistency term *α^E^*(Π_P_***u***, Π_P_***u***) can be estimated using *s*^E^ as follows:

(51) $$s^{E}\left(\Pi_{P}u,\Pi_{P}u\right)={\bar{d}}^{T}\left[\eta^{E}P_{u}^{T}P_{u}\right]\bar{d}$$

The exact solution of *α^E^*(Π_P_***u***, Π_P_***u***) is given in Equation (48). Equating the traces of the two matrices, *η*^E^ is determined:

(52) $$\eta^{E}=\frac{trace(SP_{c}^{T}DP_{c})}{trace(P_{u}^{T}P_{u})}$$

Combining Equations (48) and (49), we have

(53) $$\alpha^{E}\left(u,u\right)={\bar{d}}^{T}\left[SP_{c}^{T}DP_{c}\right]\bar{d}+{\bar{d}}^{T}\left[\eta^{E}{\left(I-P_{u}\right)}^{T}\left(I-P_{u}\right)\right]\bar{d}$$

Mimicking what we did for the bilinear form *α^E^*, the bilinear forms *α^M^* and *α^V^* are formulated as

(54) $$\alpha^{M}\left(u,u\right)={\bar{d}}^{T}\left[\frac{2\rho }{\Delta^{2}}H^{T}P_{m}H\right]\bar{d}+{\bar{d}}^{T}\left[\eta^{M}{\left(I-P_{u}\right)}^{T}\left(I-P_{u}\right)\right]\bar{d}$$

and

(55) $$\alpha^{V}\left(u,V_{0}\right)={\bar{d}}^{T}\left[\frac{2\rho }{\Delta }H^{T}P_{m}H\right]V_{0}+{\bar{d}}^{T}\left[\eta^{V}{\left(I-P_{u}\right)}^{T}\left(I-P_{u}\right)\right]V_{0},$$

respectively. Here, ***P****_m_* is a 2*n* × 2*n* constant matrix as

(56) $$P_{m}=\iint_{\Omega }{\left(T^{*}\right)}^{T}T^{*}dxdy,$$

(57) $$\eta^{M}=\frac{2\rho }{\Delta^{2}}\frac{trace(H^{T}P_{m}H)}{trace(P_{u}^{T}P_{u})}$$

and

(58) $$\eta^{V}=\frac{2\rho }{\Delta }\frac{trace(H^{T}P_{m}H)}{trace(P_{u}^{T}P_{u})}.$$

The integrations in Equation (56) can be exactly calculated by using the simplex integration. Now, we have successfully formulated all bilinear forms in Equation (43).

Then, the linear forms *f^σ^*, *f^b^*, and *f^t^* in Equation (44) are investigated. Obviously, *f^t^* can be computed directly. Because ***u*** is linear on each edge of Ω, a definite shape function matrix ***N***(***x***) can be easily determined for a point ***x*** on ∂Ω to compute ***u***(***x***) using Equation (6). Thus, we have

(59) $$f^{t}={\bar{d}}^{T}\int_{\partial \Omega }N^{T}\left(x\right)t\;d\Gamma_{t}$$

Only the constant stress associated with Π_P_***u*** can be valued in the first-order VEM, so ***σ***_0_ is a constant stress in this study. Using the integration by parts, the linear form *f^σ^* can be processed as

(60) $$f^{\sigma }=\int_{\Omega }\varepsilon^{T}\left(u\right)\sigma_{0}\;dx={\bar{d}}^{T}\left(SP_{c}^{T}\sigma_{0}\right)$$

Treating the constant body force ***b***(*x*, *y*) as an element in ***P_1_***(Ω), we have

(61) $$f^{b}=\int_{\Omega }u^{T}b\;dx=\int_{\Omega }{\left(\Pi_{P}u\right)}^{T}b\;dx={\bar{d}}^{T}\left[H^{T}\int_{\Omega }{\left(T^{*}\right)}^{T}\;dx\right]b={\bar{d}}^{T}{\left(L^{b}\right)}^{T}b$$

in which ***L****^b^* is a 2 × 2 *n* matrix as follows:

(62) $$L^{b}=\left[\begin{array}{ccccccc} L_{1}^{b} & 0 & L_{2}^{b} & 0 & \ldots & L_{n}^{b} & 0\\ 0 & L_{1}^{b} & 0 & L_{2}^{b} & \ldots & 0 & L_{n}^{b} \end{array}\right]$$

and

(63) $$L_{i}^{b}=\frac{S}{n}+0.5\left(\begin{array}{cc} l_{i}n_{ix}+l_{i+1}n_{(i+1)x} & l_{i}n_{iy}+l_{i+1}n_{(i+1)y} \end{array}\right)\left(\begin{array}{c} x_{0}-x^{*}\\ y_{0}-y^{*} \end{array}\right)$$

So far, all bilinear and linear forms in Equations (4) and (8) were formulated. The energy induced by the contact constraints are investigated in next section.

### 4.4. Computation of **J**’(v) Due to the Contact Constraints

In the block system, no tension and interpenetration is allowable when the collision of blocks occurs. Three contact states, “open”, “lock”, and “slide”, are defined in DDA. By adopting stiff springs, different contact constraints are imposed according to the state.

**Figure 5:**
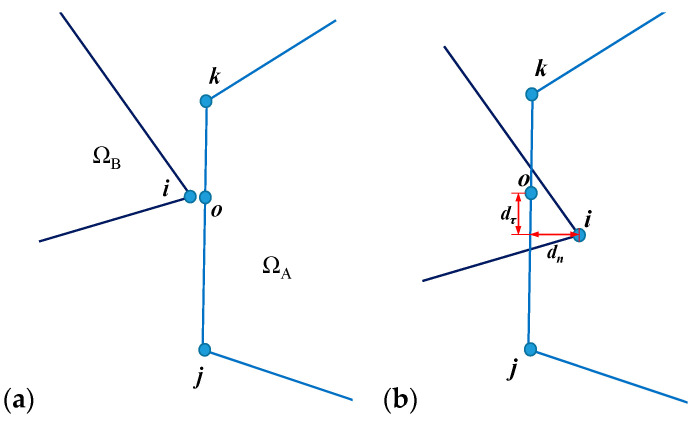
A typical contact pair at (**a**) the beginning and (**b**) the end of the current time step.

A typical contact pair that is composed by a vertex *i* of Ω*_B_* and an entrance line *jk* of Ω*_A_* is plotted in Figure 5a. Point *o* is the closest point from line *jk* to point *i*. At the start of a time step, the coordinates of points *i*, *j*, *k*, and *o* are (*x_i_*, *y_i_*), (*x_j_*, *y_j_*), (*x_k_*, *y_k_*), and (*x_o_*, *y_o_*), respectively. Within this time step, their displacements are (*u_i_*, *v_i_*), (*u_j_*, *v_j_*), (*u_k_*, *v_k_*), and (*u_o_*, *v_o_*), respectively. Then, the normal penetration *d**_n_*** and tangential relative displacement *d**_τ_*** in Figure 5b can be measured using their new coordinates when the time step terminates.

If *d**_n_*** has a negative value, the contact pair must be “open” and no contact constraint applies, which results in no spring being added into the block system. Otherwise, a normal stiffness spring is added to oppose the penetration, which results in a deformation energy *J****^n^***. Suppose that the two blocks contact each other along a coincident edge. The Coulomb friction law is adopted to determine whether the tangential relative motion is permitted. If the tangential relative motion is inadmissible, the contact pair is “lock” and a stiffness spring along the tangential direction is introduced to resist the tangential relative motion, leading to a deformation energy *J****^τ^***. Otherwise, the contact pair is “slide” and a pair of friction forces is adopted along the tangential direction, inducing a potential energy *J****^f^***. The allowable upper bound of the Coulomb friction law is considered the value of the friction forces. For the block system, ***J***’(v) in Equation (1) can be obtained by collecting *J****^n^***, *J****^τ^*,** and *J****^f^*** of all contact pairs.

In practice, the states of all contact pairs have to be supposed in advance, and then, the “open-close” iteration is executed to guarantee the validity of the states. First, assemble the global equations based on the assumed contact states and obtain an interim displacement solution. Next, check the states of the contact pairs by using the interim displacement solution and alter the previously supposed states. Modify the contact constraints concerning every contact pair. Then, renew and resolve the global equations to obtain an updated interim displacement solution. Finally, stop the iteration when the actual states of all contact pairs agree with the supposed states and acquire the ultimate displacement solution.

Firstly, considering that the displacement should be small in a time step, the normal penetration *d**_n_*** has an approximation (61) by neglecting the second-order infinitesimal value:

(64) $$d_{n}=-\left[\frac{S_{0}}{l}+\frac{1}{l}\left(\begin{array}{cc} y_{j}-y_{k} & x_{k}-x_{j} \end{array}\right)\left(\begin{array}{c} u_{i}\\ v_{i} \end{array}\right)+\frac{1}{l}\left(\begin{array}{cc} y_{k}-y_{i} & x_{i}-x_{k} \end{array}\right)\left(\begin{array}{c} u_{j}\\ v_{j} \end{array}\right)+\frac{1}{l}\left(\begin{array}{cc} y_{i}-y_{j} & x_{j}-x_{i} \end{array}\right)\left(\begin{array}{c} u_{k}\\ v_{k} \end{array}\right)\right]$$

in which

(65) $$S_{0}=\left|\begin{array}{ccc} 1 & x_{i} & y_{i}\\ 1 & x_{j} & y_{j}\\ 1 & x_{k} & y_{k} \end{array}\right|,l=\sqrt{{\left(x_{j}-x_{k}\right)}^{2}+{\left(y_{j}-y_{k}\right)}^{2}}$$

Given that the stiffness of the normal spring is *ρ**_n_***, the normal spring resisting the normal penetration induces the deformation energy as follows:

(66) $$J^{n}=0.5\rho_{n}d_{n}^{2}$$

Minimization of *J**^n^*** causes three second-order vectors to be added to the global load vector and nine second-order square matrices to be added into the global stiffness matrix.

Then, similar to *d**_n_***, the tangential relative displacement *d**_τ_*** can be formulated as

(67) $$d_{\tau }=\frac{{\bar{S}}_{0}}{l}+\frac{1}{l}\left(\begin{array}{cc} x_{k}-x_{j} & y_{k}-y_{j} \end{array}\right)\left(\begin{array}{c} u_{i}\\ v_{i} \end{array}\right)+\frac{\mu }{l}\left(\begin{array}{cc} x_{j}-x_{k} & y_{j}-y_{k} \end{array}\right)\left(\begin{array}{c} u_{j}\\ v_{j} \end{array}\right)+\frac{1-\mu }{l}\left(\begin{array}{cc} x_{j}-x_{k} & y_{j}-y_{k} \end{array}\right)\left(\begin{array}{c} u_{k}\\ v_{k} \end{array}\right)$$

where

(68) $${\bar{S}}_{0}=\left(\begin{array}{cc} x_{k}-x_{j} & y_{k}-y_{j} \end{array}\right)\left(\begin{array}{c} x_{i}-x_{0}\\ y_{i}-y_{0} \end{array}\right),\mu =\frac{1}{l}\left(\sqrt{{\left(x_{0}-x_{k}\right)}^{2}+{\left(y_{0}-y_{k}\right)}^{2}}\right)$$

Provided that the stiffness of the tangential spring is *ρ**_τ_***, the tangential spring results in the deformation energy:

(69) $$J^{\tau }=0.5\rho_{\tau }d_{\tau }^{2}$$

Minimization of *J**^τ^*** also leads to three second-order vectors being added to the global load vector and nine second-order square matrices being added into the global stiffness matrix.

Finally, if the contact pair is “slide”, a pair pf friction forces is imposed along the tangential direction instead of the tangential spring. The unit direction vector from point *j* to point *k* is

(70) $$\tau =\frac{1}{l}{\left(\begin{array}{cc} \left(x_{k}-x_{j}\right) & \left(y_{k}-y_{j}\right) \end{array}\right)}^{T}$$

The allowable upper bound of the Coulomb friction law is denoted by *f_up_*. For *d**_τ_*** in Figure 5b, the friction force *f_up_**τ*** acts on Ω*_B_* at point *i* and the friction force—*f_up_**τ*** acts on Ω*_A_* at point *o*. The potential energy caused by the pair of friction force can be formulated as

(71) $$J^{f}=-\mu \left(\begin{array}{cc} u_{j} & v_{j} \end{array}\right)f_{up}\tau -\left(1-\mu \right)\left(\begin{array}{cc} u_{k} & v_{k} \end{array}\right)f_{up}\tau +\left(\begin{array}{cc} u_{i} & v_{i} \end{array}\right)f_{up}\tau$$

Minimization of *J**^f^*** causes three second-order vectors to be added to the global load vector. It is noticed that the Coulomb model of friction assumes that the sliding frictional force is proportional to the normal contact force. Provided that the joints between blocks have the mechanical properties of the friction angle *φ* and the inner cohesion *c*, *f_up_* is equivalent to |*ρ**_n_**d**_n_***|tan*φ* + *c*. If Equation (64) is adopted to estimate *f_up_*, the frictional energy term will contribute not only to the global force vector but also to the stiffness matrix, leading to non-symmetry of the stiffness matrix. To avoid this issue, *f_up_* is estimated by using *d**_n_*** obtained in the previous “open–close” iteration. In the view that the variation in *d**_n_*** is small when the “open–close” iteration is about to converge, such a simplification is acceptable.

In the original DDA, the incremental vertex displacements are estimated using Equation (11), while in the proposed method, the incremental vertex displacements are selected as the degrees of freedom. Therefore, the contributions of the contact restraints to the global stiffness matrix and load vector have simpler but more precise expressions in the proposed method than in the original DDA [36].

Minimize the potential energy-induced contact pairs in the block system one by one, and the contribution of ***J***’(v) to the global equations are obtained and Equation (9) is fulfilled.

When Equation (9) is fulfilled completely, the global equations are resolved as the traditional DDA. For one time step, the vertex coordinates are updated by adding their incremental displacements to the previous coordinates once the open–close iteration converges. In addition, the constant stress associated with accumulation of the constant strain in Π*_P_**u*** over the foregoing time steps is considered the initial stress for the following time step.

## 5. Numerical Examples

Some numerical examples are analyzed to test the proposed method in this section. To evaluate the correctness, precision, and efficiency of the new approach, DDA with the post-adjustment strategy and vertex displacements-based DDA based on Wachspress interpolation [36] are adopted to solve these examples. For an objective comparison on the computational efficiency, all computations are conducted on the same laptop with 8 GB of RAM and Intel Core i7-6500U CPU. When conducting contact computation, *ρ**_n_*** was prescribed as 100*E* and *ρ**_τ_*** was prescribed as 40E for all methods. Here, *E* is Young’s modulus of the block.

### 5.1. Rotating Triangular Block Problem

**Figure 6:**
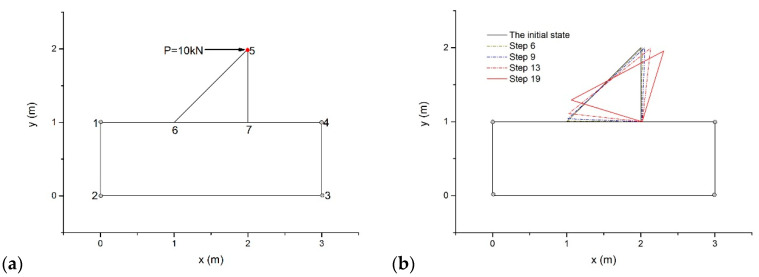
(**a**) A triangular block on the foundation and (**b**) its movement under a horizontal point load.

As drawn in Figure 6a, a rotating triangular block problem was designed by the authors to investigate the contact states in the equilibrium equation and in the resulting configurations. There was a triangular block on a rectangular ramp. The block and the ramp had the same material properties: *ρ* = 2.8 g/cm^3^, *E* = 20 GPa, and Poisson’s ratio *ν* = 0.25. The gravity force of this model was ignored, and a concentrated load *P* = 10 kN acted at point *5* along the horizontal direction. All vertices of the foundation were fixed as the boundary conditions.

Firstly, DDA with the post-adjustment strategy was adopted to simulate the dynamic behavior of the model by using the max step displacement ratio *δ* = 0.1 and Δ = 0.01 s. Figure 6b illustrates the configurations at the end of the 6, 9, 13, and 19 time steps. The triangular block rotated around point *7* for the eccentric moment from *P*. In the model, edge *14* had no rotation for its two end points that were both fixed. For the contact pair point *7*–edge *14*, we noted the normal penetration value in the equilibrium equations and measured the normal penetration value in the updated configurations. The results are listed in Table 1.

Although the rotational angle *r_0_* is very tiny in each time step, the difference is sometimes considerable in the two normal penetration values. Moreover, the normal penetration value is negative in the resulting configurations at steps 5, 6, and 7, which violates the actual contact conditions completely.

Then, the example was reanalyzed using the proposed method. For the contact pair point *7*–Edge *14*, the normal penetration values are also given in Table 1. The results indicated that the two normal penetration values are identical when the incremental displacements at the vertices are directly selected as the degrees of freedom.

Finally, vertex displacement-based DDA based on Wachspress interpolation [36] was used to solve this example and produced the same results as the proposed method. In this example, the time consumed by DDA with the post-adjustment strategy was 0.034 s, vertex displacement-based DDA in [36] spent 0.038 s, and the proposed method consumed 0.037 s.

### 5.2. Sliding Problem

**Figure 7:**
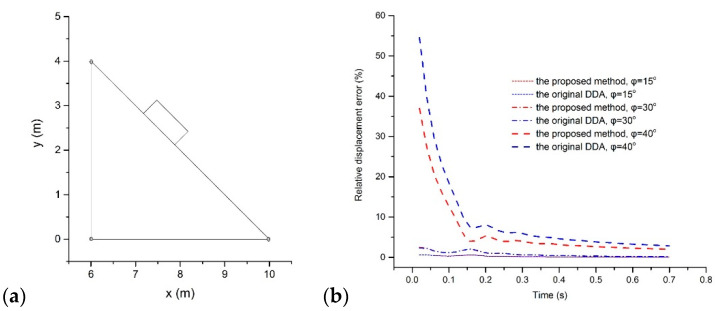
(**a**) The configuration of the sliding problem and (**b**) the relative displacement errors of the proposed method and DDA with the post-adjustment strategy.

There was a rectangular block on a triangular ramp, as Figure 7a shows. The top surface of the ramp had a slope angle of 45°. The block and ramp were prescribed to have the same mechanical parameters: *ρ* = 2.5 × 10^3^ kg/m^3^, *E* = 35 MPa, and *ν* = 0.3. The external load only came from the gravitational force with gravity acceleration *g* = 9.8 m/s^2^. No inner cohesion was involved in this model. All vertices of the ramp were fixed as the boundary conditions.

The block slides along the ramp if the friction angle of the interface *φ* is less than 45°, and its displacement is

(72) $$s=0.5g(\sin45^{o}-\tan\varphi \cos45^{o})t^{2}$$

in which *s* denotes the sliding distance (m) of the rectangle and *t* is the elapsed time (s).

To appraise the accuracy of the new method, three values, 15°, 30°, and 40°, were assigned to the friction angle. Taking Δ = 0.02s and *δ* = 0.10, 35 time steps were computed. Referring to the analytical solutions of Equation (72), the relative errors of the proposed approach and the original DDA results were measured and are drawn in Figure 7b.

Above all, the relative errors of the two methods both appear to be correlated with the friction angle. Large friction angles always induce larger errors than small angles. The reason behind that is the approximation strategy of the contact force adopted in DDA. For a new contact pair, the proposed method initializes the contact force to 0 as the classical DDA and approximates the exact value according to the resulting interpenetration or inconsistent tangential motion between blocks.

Then, the relative errors of the two methods both rapidly drop off as the time steps increase. In the cases *φ* = 15° and *φ* = 30°, the displacement differences in the two methods are quite small. Both methods induced a relative error decreasing from 0.65% to 0.06% when *φ* = 15° and from 2.4% to 0.2% when *φ* = 30°, making it difficult to distinguish their relative error curves in Figure 7b. When *φ* = 40°, the original DDA results in a relative error of 55% at the first time step and a relative error of 2.9% at the end while the new method leads to a relative error of 37% at the first time step and a relative error of 2.0% at the end. Therefore, the proposed method provides higher accuracy results for this problem than the original DDA.

**Figure 8:**
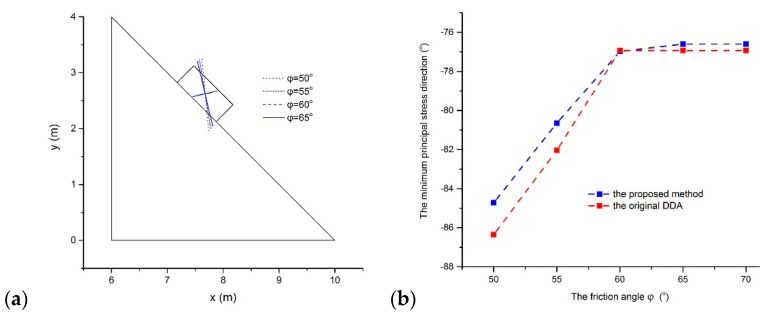
(**a**) The variations in minimum principal stress direction with the friction angles and (**b**) the difference in minimum principal stress direction of the two methods.

As in the original DDA, the constant stress is estimated using the proposed method because only the constant strain associated with Π*_P_**u*** can be determined within the block. To investigate the difference in stress results of DDA with the post-adjustment strategy and the proposed method, the sliding problem was reanalyzed. Because the stress is unstable in a dynamic analysis, five friction angles, 50°, 55°, 60°, 65°, and 70° were adopted and static analysis with 50 time steps was executed to obtain a stable stress result. Firstly, little difference occurs in the resulting stress values of the two methods. When *φ* = 50°, the maximal principal stress *σ*_1_ = 0.50 kPa and the minor principal stress *σ*_2_ = −6.91 kPa in the original DDA and *σ*_1_ = 0.63 kPa and *σ*_2_ = −6.66 kPa in the proposed method. When *φ* = 70°, *σ*_1_ = 2.22 kPa and *σ*_2_ = −5.97 kPa in the original DDA and *σ*_1_ = 2.22 kPa and *σ*_2_ = −5.91 kPa in the proposed method. Then, the minimum principal stress direction appears to increase gently with the friction angle increasing, as shown in Figure 8a, and it stabilizes when the friction angle increases to 65°. The variations in the minimum principal stress direction with the friction angles are plotted for the two methods in Figure 8b. The proposed method has a gentler direction for the minimum principal stress. The stable direction for the minimum principal stress is −76.93° in the original DDA and −76.60° in the proposed method.

For this problem, the calculation times consumed by the three methods appear to have a clear gap. Taking the case *φ* = 30° as an example, DDA with the post-adjustment strategy took 0.041 s. The time consumed by vertex displacement-based DDA in [36] is 0.108 s. This is because numerical integrations is necessary in computation for a rectangular block when using vertex displacement-based DDA based on Wachspress interpolation. However, only 0.042 s is consumed by vertex displacement-based DDA using VEM.

### 5.3. Surrounding Rock Problem

**Figure 9:**
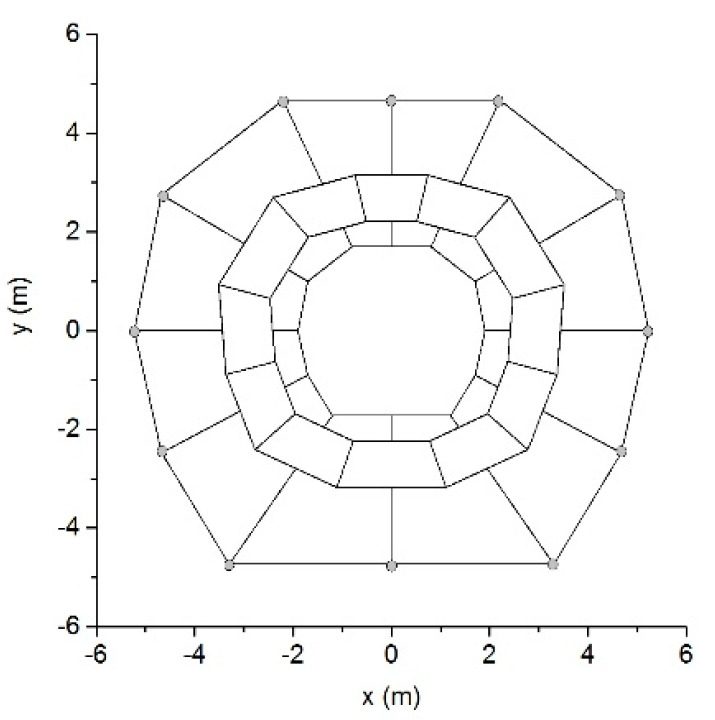
A surrounding rock model.

The stability assessment of a surrounding rock is an important problem for the design and construction of underground engineering. Figure 9 describes a surrounding rock model composed of 36 rock blocks. Both convex and concave blocks were included in this model. All blocks were assumed to have the same mechanical characteristics: *ρ* = 2.8 g/cm^3^, *ν* = 0.20, and *E* = 200 MPa. Only the gravitational force with *g* = 10 m/s^2^ acts on the model. The hoop joints in the surrounding rock divided the model into three layers. The outermost boundaries of this model were fixed as the displacement boundary conditions.

**Figure 10:**
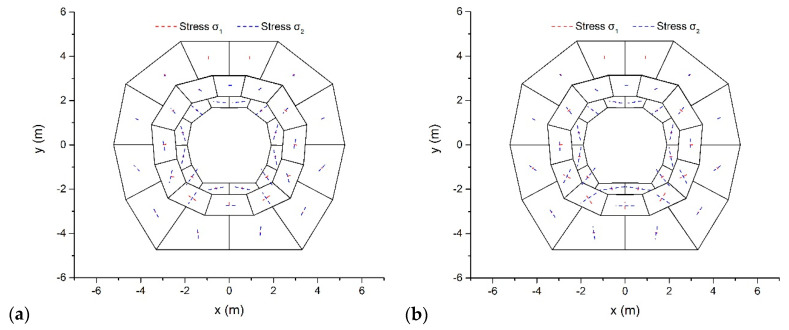
Configurations and main stresses after 100 time steps under two different friction angles: (**a**) φ = 30° and (**b**) φ = 0°.

Using *δ* = 0.005 and a self-adjusting Δ from the code, the proposed method was employed to conduct a static analysis of the problem. Taking the friction angle *φ* = 30° for the joints between blocks, Figure 10a illustrates the ultimate configurations and main stresses after 100 time steps. The proposed method arrived at the conclusion that the model is stable. The stress level decreases from the outer layer to the inner layer. At the top and the bottom, the inner layer suffers the largest horizontal compress effect. The top two rocks in the inner layer suffer *σ*_1_ = −0.4 kPa and *σ*_2_ = −109.7 kPa, which induces a minimum principal stress direction of 6.7°. The bottom two rocks in the inner layer suffer *σ*_1_ = −15.6 kPa and *σ*_2_ = −138.6 kPa, and the direction of the minimum principal stress is 11.4°.

Considering the mechanical property of the joints may be damaged by the blasting excavation effect, friction was neglected and this model was reanalyzed. Figure 10b plots the results after 100 time steps, which adopts the same tags for stress vectors as Figure 10a. Because of the arch effect, the surrounding rock is also stable and the stress level still decreases from the outer to the inner layers. In contrast, the stress values appear to increase in the case of no friction. The top two rocks in the inner layer suffer *σ*_1_ = −6.2 kPa and *σ*_2_ = −117.6 kPa, and the minimum principal stress direction is 9.2°. The bottom two rocks in the inner layer suffer *σ*_1_ = −24.8 kPa and *σ*_2_ = −227.5 kPa, which has a minimum principal stress direction of 8.4°. The reason for this is that the stability of the arch only relies on the compression between blocks when the joints friction effect is absent.

The stability of the surrounding rock in this problem was also verified by using DDA with the post-adjustment strategy. Vertex displacement-based DDA based on Wachspress interpolation [36] fails to solve this problem because Wachspress interpolation does not apply for concave blocks. For this problem, more calculation time is consumed by the proposed method than DDA with the post-adjustment strategy, which can be attributed to the fact that the proposed method almost doubles DDA with the post-adjustment strategy in the dimension of the total degrees of freedom. Taking the case with no friction as an example, DDA with the post-adjustment strategy took 0.891 s and the proposed method took 1.117 s.

### 5.4. Block Wall Failure Problem

**Figure 11:**
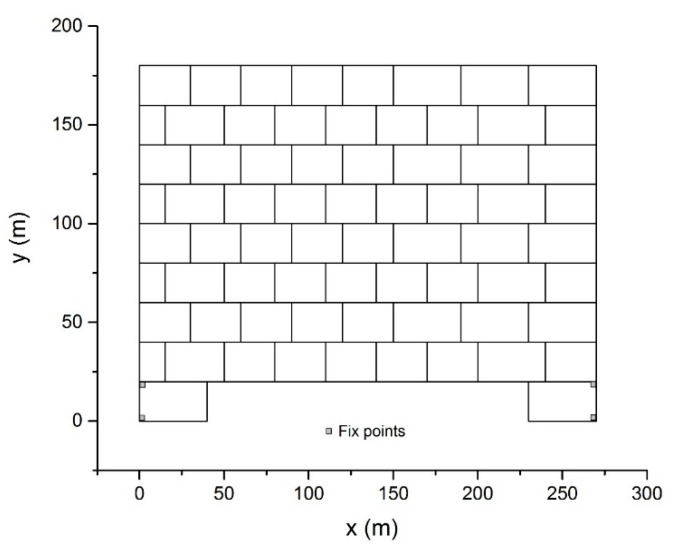
The block wall model.

As Figure 11 shows, a wall containing 70 blocks was analyzed to test the capacity of the proposed method. The wall had a width of 270 m and a height of 180 m. The mechanical parameters of the blocks were given as *ρ* = 2.0 × 10^3^ kg/m^3^, *E = 500* MPa, and *ν* = 0.25. The external load only included the gravitational force with *g* = 10 m/s^2^. No friction occurred between the blocks. The outer vertices of two base blocks were fixed as the boundary conditions.

**Figure 12:**
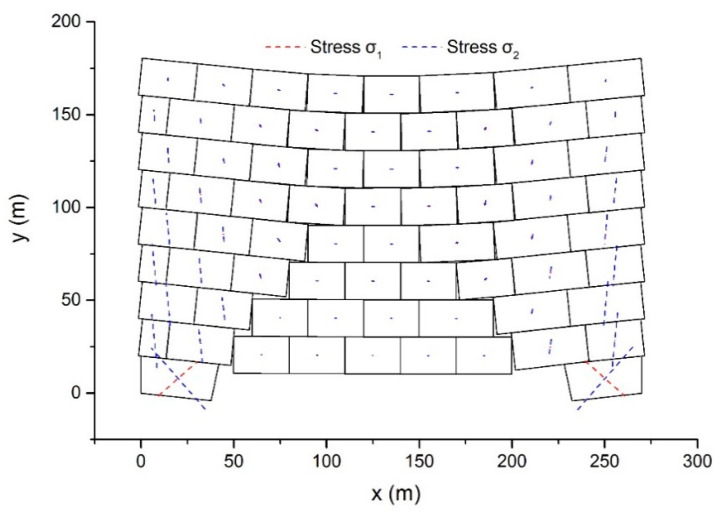
The configurations and main stresses after 7 s using the proposed method.

Taking Δ = 0.012 s and *δ* = 0.01, the dynamic behavior of the model was simulated. Figure 12 demonstrates the resulted configurations and main stresses after 7 s.

First, at the bottom, the central blocks descend because of the absence of supports. Next, the middle blocks subside one layer after another. Simultaneously, the movement of the middle blocks leads to rotation and inclination of the left and right blocks. Submitting to the gravitational force of the upper blocks, the bottom two blocks become deformed. After 7 s elapsed, the top boundary of the model appears to be a gentle curve. The stress value of the blocks in the middle is rather small compared to the blocks in other regions, and the two bottom blocks suffer the largest stress. In the bottom right block, the main stresses are *σ*_1_ = 560 kPa and *σ*_2_ = −880 kPa and the direction of the minimum principal stress is −48°.

**Figure 13:**
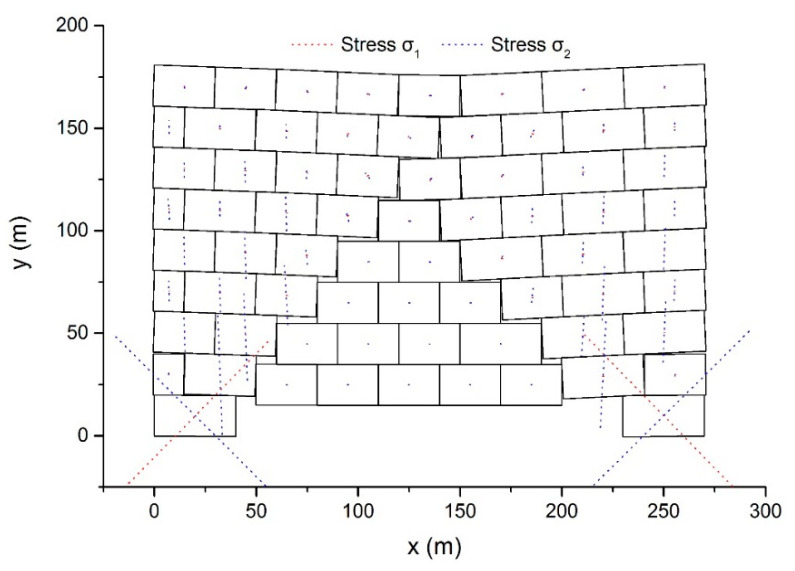
The configurations and main stress vectors after 7 s by DDA with the post-adjustment strategy.

With Δ and *δ* unchanged, the dynamic behavior of the example was reanalyzed using DDA with the post-adjustment strategy. The configurations and main stress vectors after 7 s elapsed are shown in Figure 13.

The difference in the results of the proposed method and DDA with the post-adjustment strategy is distinct. The two bottom blocks suffer larger stresses in DDA with the post-adjustment strategy than in the proposed method. In the bottom right block, *σ*_1_ = 2115 kPa and *σ*_2_ = −2205 kPa, and the minimum principal stress has a direction of −44°. Rotation and inclination of the blocks in the left and right regions are smaller compared to the results of the proposed method. The difference in the displacement mode of the two methods has great effects on the difference between the results. The linear displacement function was adopted directly in DDA with the post-adjustment strategy. However, in the case when the vertex number was *n* > 3, the contribution of the higher-order displacement was added to the proposed method. Therefore, the proposed approach provides a block of *n* > 3 vertices with larger deformability than DDA with the post-adjustment strategy. Once the deformation of this block is not restrained by the adjacent blocks, a larger deformation occurs in the proposed method.

For this problem, DDA with the post-adjustment strategy took 1.606 s, the new method took 1.943 s, and vertex displacement-based DDA based on Wachspress interpolation [36] took 4.762 s.

The results of Examples 1 and 2 verified that the proposed method has a higher accuracy in the contact computation than DDA with the post-adjustment strategy. Previous studies to construct vertex displacement-based DDA focused on governing the displacement of a block using the displacement functions in PFEM, e.g., Wachspress interpolation [36]. The issue that Wachspress interpolation does not apply for concave blocks can be remedied by introducing those generalized barycentric coordinates well-defined in the concave domain. If the displacement functions in PFEM are adopted in the construction of vertex displacement-based DDA, the numerical integrations have to be executed for a block with more than three vertices, inducing lower computational efficiency for large-scale problems. Relatively speaking, the proposed method may be a better choice when taking the displacements at the vertices as the degrees of freedom. In the view that the degrees of freedom have a larger dimension in the proposed method than in the original, the new method has a lower computational efficiency than the original DDA. However, a 2% to 20% increase in time consumption is achieved, which is acceptable.

## 6. Conclusions

In this study, the increments of displacements at block vertices in one time step were selected as the new degrees of freedom for a block. The virtual element spaces were defined for a block and the block system. In the framework of VEM theory, the total potential energy of the DDA block system was estimated and minimized to obtain the global equilibrium equation and load vector. The proposed method can be considered an improvement of DDA by reformulating the block displacement using VEM and can be regarded as an attempt to model the dynamic behavior of the block system using VEM coupled with the contact theory in DDA.

The proposed method has some advantages. On the one hand, the approximation of cos *r_0_* and sin *r_0_* in the displacement function of the original DDA is avoided in the proposed method. The degrees of freedom are directly added to the former coordinates in the renewal of the vertex coordinates, which induces a simpler expression regarding the contact restraint impositions. On the other hand, the contact detection and the contact theory can still be adopted as in the original DDA. Meanwhile, most of the numerical integrations are executed along the block boundary when calculating the global stiffness matrix and load vector. Only numerical integrations within the block can be conducted using the simplex integration as in the classical DDA.

Additionally, ***V***_1_(Ω) is taken as the virtual element space for a block in this study. If the stress variability within the block is expected, a higher-order virtual element space instead of ***V***_1_(Ω), e.g., ***V***_2_(Ω) and ***V***_3_(Ω), can be adopted for a block. When using a higher-order virtual element space, the degrees of freedom should be augmented; refer to [46]. Although this study is limited to 2D analysis, the method to improve DDA by reformulating the block displacement using VEM can be mimicked in a 3D case.

## Figures and Tables

**Table 1:** The penetrations of the contact pair point 7–edge 14 by DDA with the post-adjustment strategy and the new method.

Time Step	The Original DDA with the Post-Adjustment Strategy		The New Method		The Rotational Angle *r_0_*
	Penetrations in the Equilibrium Equations (m)	Penetrations in the Updated Configurations (m)	Penetrations in the Equilibrium Equations (m)	Penetrations in the Updated Configurations (m)	
1	8.3289 × 10^−^^9^	8.3288 × 10^−^^9^	8.32890 × 10^−^^9^	8.32890 × 10^−^^9^	−0.000001
2	2.5026 × 10^−^^8^	2.5026 × 10^−^^8^	2.50260 × 10^−^^8^	2.50260 × 10^−^^8^	−0.000000
3	7.4918 × 10^−^^8^	7.4918 × 10^−^^8^	7.49180 × 10^−^^8^	7.49180 × 10^−^^8^	−0.000000
4	3.3772 × 10^−^^7^	1.4587 × 10^−^^7^	3.37720 × 10^−^^7^	3.37720 × 10^−^^7^	−0.001073
5	1.0094 × 10^−^^6^	−7.2045 × 10^−^^7^	1.00970 × 10^−^^6^	1.00970 × 10^−^^6^	−0.003218
6	3.0067 × 10^−^^6^	−1.8379 × 10^−^^6^	3.01240 × 10^−^^6^	3.01240 × 10^−^^6^	−0.005375
7	8.9005 × 10^−^^6^	−7.2391 × 10^−^^7^	8.92310 × 10^−^^6^	8.92310 × 10^−^^6^	−0.007554
8	2.6114 × 10^−^^5^	9.8896 × 10^−^^6^	2.62290 × 10^−^^5^	2.62290 × 10^−^^5^	−0.009768
9	7.5032 × 10^−^^5^	5.0108 × 10^−^^5^	7.54400 × 10^−^^5^	7.54400 × 10^−^^5^	−0.012047
10	2.0631 × 10^−^^4^	1.7005 × 10^−^^4^	2.07880 × 10^−^^4^	2.07880 × 10^−^^4^	−0.014444
11	4.9419 × 10^−^^4^	4.4322 × 10^−^^4^	4.97090 × 10^−^^4^	4.97090 × 10^−^^4^	−0.017006
12	7.6165 × 10^−^^4^	6.9448 × 10^−^^4^	7.63320 × 10^−^^4^	7.63320 × 10^−^^4^	−0.019372
13	6.5260 × 10^−^^4^	5.7022 × 10^−^^4^	6.50170 × 10^−^^4^	6.50170 × 10^−^^4^	−0.021275
14	3.2463 × 10^−^^4^	2.2251 × 10^−^^4^	3.22710 × 10^−^^4^	3.22710 × 10^−^^4^	−0.023478
15	2.6022 × 10^−^^4^	1.2772 × 10^−^^4^	2.68970 × 10^−^^4^	2.68970 × 10^−^^4^	−0.026494
16	5.7111 × 10^−^^4^	3.9996 × 10^−^^4^	5.96770 × 10^−^^4^	5.96770 × 10^−^^4^	−0.029810
17	9.6448 × 10^−^^4^	7.5227 × 10^−^^4^	9.91470 × 10^−^^4^	9.91470 × 10^−^^4^	−0.032847
18	9.2970 × 10^−^^4^	6.7950 × 10^−^^4^	9.13440 × 10^−^^4^	9.13440 × 10^−^^4^	−0.035286
19	3.3880 × 10^−^^4^	4.7618 × 10^−^^5^	2.94330 × 10^−^^4^	2.94330 × 10^−^^4^	−0.037664

## Data Availability

The data used to support the findings of this study are available from the corresponding author upon request.
